# Who Establishes the Presence of a Mental Disorder in Defendants? Medicolegal Considerations on a European Court of Human Rights Case

**DOI:** 10.3389/fpsyt.2017.00199

**Published:** 2017-10-16

**Authors:** Tijs Kooijmans, Gerben Meynen

**Affiliations:** ^1^Tilburg Law School, Tilburg University, Tilburg, Netherlands; ^2^Faculty of Humanities, VU University Amsterdam, Amsterdam, Netherlands

**Keywords:** legal insanity, mental disorder, forensic psychiatric evaluation, behavioral experts, European Court of Human Rights

## Abstract

Legal insanity is a peculiar element of criminal law, because it brings together two very different disciplines: psychiatry and psychology on the one hand and the law on the other. One of the basic questions regarding evaluations of defendants concerns the question of who should establish “true mental disorder,” the judge or the behavioral expert? This question is complicated, and in this contribution it will be explored based on a Dutch case that was eventually decided by the European Court of Human Rights (ECtHR). We will argue that the ECtHR provides a valuable legal framework. Based on its merits, the framework could also be of interest to countries outside the Court’s jurisdiction.

## Introduction

In many legal systems, a person who commits an offense can be held criminally liable. Criminal liability is based on the assumption that the offender is to blame for his criminal behavior because he had freedom of action and the possibility not to break the law. Reversely, a very prominent principle of criminal law is that a person cannot be punished for an offense if he is not to blame for what he did: no punishment without blame (*nulla poena sine culpa*). Usually, it is considered as an exception not to hold a person criminally responsible if he committed an offense (1–3). This exceptional circumstance may be rooted in a mental disorder which influenced the perpetration of the crime in such a way that the judge cannot hold the offender liable. In fact, there are very different ways of substantiating insanity in domestic criminal law systems (2).

In many legal systems, legal insanity is a defense which has to be raised by the defendant. For instance, in most states in the US, insanity is a defense.^1^ If the defense is raised by the defendant himself, he is very likely to cooperate with a behavioral evaluation. Meanwhile, in other systems, insanity assessments may be court ordered, or ordered by the prosecution. In such jurisdictions, it is much less clear that the defendant will cooperate with the evaluation. The Netherlands is a system in which behavioral evaluations—which may lead to the assessment of insanity—are, in the standard situation, ordered by the prosecution or the judge (5). We discuss a case in which the evaluation was ordered by a Court and in which the defendant refused to cooperate. Even though neither the psychiatrist nor the psychologist could establish a diagnosis, the Court nevertheless decided that the defendant was suffering from a mental illness and his criminal responsibility was therefore considered diminished. Moreover, he was sentenced to a hospital order (TBS; forensic psychiatric care, see below). The decision was appealed, and, eventually, the European Court of Human Rights (ECtHR) decided about the case (47 member states fall under the Court’s jurisdiction).

The question of who should decide about the presence of a mental illness is not new (6), in fact there has been a “longstanding and widespread concern that,” as Buchanan (7) (p. 19) writes, “psychiatric testimony is more likely than other evidence to intrude into the jury’s realm.” This was especially relevant regarding the Product test, in which the presence of a mental illness, diagnosed by the behavioral expert, appeared to almost immediately result in insanity. This was considered undesirable, and in fact a “psychiatrization of criminal law” Gerber (8) (p. 125). In the US, the courts do not have to rely on a behavioral expert’s advice when deciding about mental abnormality, as Morse (9) (p. 894, references omitted) explains:

“The criminal law can, but need not, turn to scientific or clinical definitions of mental abnormality as legal criteria when promulgating mental health laws. The Supreme Court has reiterated on numerous occasions that there is substantial dispute within the mental health professions about diagnoses, that psychiatry is not an exact science, and that the law is not bound by extra-legal professional criteria. The law often uses technical terms, such as ‘mental disorder,’ or semi-technical qualifiers, such as ‘severe,’ but non-technical terms, such as ‘mental abnormality,’ have also been approved. Legal criteria are adopted to answer legal questions. As long as they plausibly do so, they will be approved even if they are not psychiatric or psychological criteria.”

Yet, what is interesting about the case we present is that the court *did turn* to behavioral experts, but when the latter were *unable* to diagnose a disorder, the Court nevertheless decided that there was a mental disorder. In fact, the Court then decided to impose the most far-reaching measure for forensic care possible in the Netherlands: TBS (see below).

The outline of the paper is as follows. First, we will further, but briefly, introduce the Dutch legal system regarding psychiatric and psychological evaluations of defendants (see Behavioral Assessment of Defendants in the Netherlands). Next, in Section “The Case: The Court of Appeal, and the ECtHR,” we present the Dutch case and we look at it in some detail (because the legal details matter), focusing on the Dutch Court of Appeal decision and the ECtRH. In Section “Analysis and Discussion,” we discuss the decisions.

## Behavioral Assessment of Defendants in the Netherlands

According to article 39 of the Dutch Criminal Code, anyone who commits an offense for which he cannot be held responsible due to a mental defect or mental disease is not punishable (5, 6, 10–12).^2^ To come to this decision, the trial judge—who does not have the expertise to assess a mental disorder of the defendant and its relation to the offense—needs to obtain information from a behavioral expert, usually a psychologist or psychiatrist. This expert advice *pro Justitia* is even a statutory requirement if the judge considers imposing a placement in a psychiatric hospital (*plaatsing in een psychiatrisch ziekenhuis*) or a hospital order (*terbeschikkingstelling, TBS)*, a criminal measure similar to the commitment order which can be imposed in the US (cf. article 330.20 lid 1 sub f New York Criminal Procedure Law). The expert advice is an important substantive safeguard with respect to the assessment by the judge of the defendant’s insanity or (diminished) responsibility.

As mentioned earlier, TBS is the most severe measure. This security measure can be imposed if the defendant, at the time of committing the (grave) offense, was suffering from a mental defect or mental disease. If the offense was directed against the physical integrity of the victim, the TBS can be extended every 2 years. In recent years, it has become more common for defendants not to cooperate with psychiatric and psychological evaluations, presumably because they fear the indeterminacy of the duration of TBS (and their lawyers may advice against cooperation for that reason). The defendant is free either to cooperate or not (5, 6). In recent years, there have been quite a few cases in which the behavioral experts were not able to do a proper evaluation of the defendant, because he did not sufficiently cooperate.^3^ Behavioral experts should not diagnose a person without performing a proper evaluation (13). In addition, the *nemo tenetur*-principle—one is not obliged to contribute to his own conviction—which can be derived from the right to a fair trial (article 6 European Convention for the Protection of Human Rights and Fundamental Freedoms, ECHR), gives the defendant the legal space to refuse to cooperate with the behavioral examination (14).

In the daily practice of Dutch criminal law, the judge explicitly asks the psychiatrist and psychologist about their opinion with regard to insanity or (diminished) criminal responsibility of the defendant. This question is part of a format that was used over the last decades in Dutch criminal law. Until recently, the behavioral expert was supposed to answer—among others—the following questions (this is the format of questions used in the case we describe):

1. Is the examined person suffering from a mental disorder/defective development of the mental faculties? If so, how can this be described diagnostically (in terms of the DSM)?
2. What was the person’s mental condition at the time the criminal offense was committed?
3. Did the mental disorder/defective development of the mental faculties influence the behavioral choices of the examined person, or his behavior during the offense, to an extent that the alleged offense can be explained from this disorder/defective development?
4. If so, can the behavioral expert substantiate:
  (a) in what way this happened,
  (b) to what extent this happened, and
  (c) which conclusion with regard to the examined person’s criminal responsibility can be advised?^4^
5. Can the behavioral expert substantiate to what extent and in what way the (possible) mental disorder/defective development of the mental faculties could lead to similar or other offenses?

The answer to question 4c by the behavioral expert used to be categorized in one out of five degrees: (i) responsibility, (ii) somewhat diminished responsibility, (iii) diminished responsibility, (iv) severely diminished responsibility, or (v) insanity (15, 16). (Currently, in the Netherlands, a three point scale is used: (1) responsibility, (2) diminished responsibility, and (3) insanity.)

Another feature of the Dutch system is that there is no formal test for legal insanity. As a result of the lack of clarity of the criteria for legal insanity, in practice, each behavioral expert creates his own frame of reference with regard to this concept (17).

In some recent cases, even though the behavioral expert could not establish any diagnosis, the judge nevertheless came to the conclusion that the defendant was suffering from a mental illness in the legal sense (18). Reversely, in a case in which the defendant was reported by a psychologist to be suffering from “hyper sexuality”—which was not described in DSM-IV-TR^5^ (Diagnostic and Statistical Manual of Mental Disorders)—the court of appeal did not impose a TBS measure because the court ruled that only mental disorders described in DSM-IV-TR could be considered a mental defect or mental disease in the legal sense. The Dutch Supreme Court overruled this decision (19). As a consequence, mental diseases that are not described in DSM-IV-TR can be considered as a mental defect or mental disease in the legal sense. However, as the Supreme Court judged without further explanation, the mere fact that a mental disease is described in DSM-IV-TR does not necessarily mean that this is a mental illness in the legal sense.^6^

With regard to the question of whether a TBS measure should be imposed, it is for the judge to decide whether the defendant was suffering from a mental illness at the time the offense was committed. According to the jurisprudence of the Supreme Court, the judge has his own responsibility with respect to this assessment and the judge is not restrained by the advice of behavioral experts (20).

One of the core activities of the judiciary is the interpretation of the (statutory) law. If security measures can be imposed to offenders who, according to the law, suffer from “a mental defect or mental disease,” it is up to the judge to interpret this legal term. The lack of a legal standard in this regard complicates this interpretation. The interpretation of the legal term “a mental defect or mental disease” should be distinguished from the question of whether the defendant actually suffers from a mental defect or disease.

## The Case: The Court of Appeal, and the ECtHR

In a famous Dutch case—the *Hoogerheide* case—the defendant was convicted to 12 years imprisonment and TBS for manslaughter of an 8-year-old boy on 1 December 2006 (18). Because the precise legal reasoning is important to our argument, in this section, we provide some crucial quotations from the Court rulings which will be discussed in the next section. The Dutch Court of Appeal judged as follows:

“If, as in the present case, the suspect has withheld his (complete) cooperation in an examination by behavioral experts, then the requirement of a (full) multidisciplinary examination within the meaning of Article 37 § 2 of the Criminal Code disappears.^7^ But the need remains for the establishment of a mental disturbance or inadequate development of the suspect’s mental faculties at the time when he committed the act. Without it, a TBS order cannot be imposed. It is up to the trial court to make that establishment. The trial court will have to let itself be guided to a very considerable extent by the findings and conclusions of behavioral experts, when the behavioral experts reach the limits of what they can take responsibility for within their scientific knowledge, the trial court will have to take its own responsibility in so far as the law gives it the necessary room. Neither statute nor case-law requires the disturbance to be classified according to the DSM-IV manual and determined by a behavioral expert. This means that, contrary to what the defense has argued, it is ultimately for the trial court, obviously with great caution, to establish the existence of a mental disturbance, even though the behavioral experts cannot reach that conclusion based on the scientific criteria and deontological standards applicable to them. The trial court will, however, have to find sufficient support for its decision in what the behavioral experts may have been able to establish and whatever other facts and circumstances may have become apparent to the trial court regarding the person of the suspect (21).”

In considering the defendant’s mental state, the Court of Appeal had regard to the following [quotation from Constancia (21)]:

– A report by a psychologist drawn up on 21 March 2004, in connection with a prosecution for armed robbery. It was noted that the applicant’s personality had not yet matured, so that it was not yet possible to find that the applicant was afflicted with an inadequate development of his mental faculties in the sense of a personality disorder. The applicant’s personality was characterized by an inadequate sense of values, a lack of fear as an inhibiting factor, impulsiveness and a tendency to overestimate himself and overlook his limitations. The danger of reoffending was considered real. The applicant’s personality development was under threat and there was a danger of further personality distortion.
– A report by a forensic psychiatrist drawn up on 4 December 2006. This reflected that the applicant behaved as if nothing could affect him and pictured himself above the situation in which he found himself as a homicide suspect; it also related some “bizarre statements” reflecting disturbed reality testing.
– A report drawn up on 21 June 2007 by a psychologist and a psychiatrist (…). It is noted that the applicant was diagnosed with a “borderline syndrome” at the age of 15 and with an “as yet immature personality with narcissistic and antisocial traits” at the age of 19. The report posited narcissistic and antisocial personality disorders, identity problems, and psychotic episodes such as would indicate the so-called borderline personality, but a schizophrenic development was not excluded. The applicant’s refusal to cooperate had made it impossible, however, to draw any definite conclusions.
– A supplementary report drawn up on 27 January 2011 by [court appointed behavioral experts] psychologist O. and psychiatrist R. Based on all the information available, including the criminal file and the audio and audio-visual recordings of interrogations, this reflected the “worrying development” of a young man who had led a detached and antisocial existence, had abused cannabis, and lived in a world of his own. As the report itself mentions, this was, in effect, the same finding as that made in 2007. The experts O. and R. were unable to supplement it with findings resulting from their own observation.^8^

In addition, the Court of Appeal made use of statements of witnesses, some of them close relatives of the applicant, and of reports by police and prison staff made after the applicant’s arrest. All described the applicant as manifesting unusual behavior. The Court of Appeal came to the conclusion that the defendant’s responsibility for the offense was diminished due to mental disturbance. Since diminished responsibility did not exclude the defendant’s accountability completely, the Court of Appeal imposed not only a TBS measure but also a prison sentence.

This judgment was upheld by the Dutch Supreme Court (22). The convicted person started proceedings at the ECtHR, complaining that the TBS measure had been imposed without objective medical expertise to support it, thus violating article 5 § 1 (e) of the ECHR, which reads as follows:

“1.Everyone has the right to liberty and security of person. No one shall be deprived of his liberty save in the following cases and in accordance with a procedure prescribed by law: (…) (e) the lawful detention (…) of persons of unsound mind.”

The ECtHR judged as follows:

‘25.The Court reiterates its established case-law according to which an individual cannot be considered to be of “unsound mind” and deprived of his or her liberty unless the following three minimum conditions are satisfied: first, he or she must reliably be shown to be of unsound mind, that is to say, a true mental disorder must be established before a competent authority on the basis of objective medical expertise; second, the mental disorder must be of a kind or degree warranting compulsory confinement; third, the validity of continued confinement depends upon the persistence of such a disorder (…).
26.Where no other possibility exists, for instance, because of a refusal of the person concerned to appear for an examination, at least an assessment by a medical expert on the basis of the file must be sought, failing which it cannot be maintained that the person has reliably been shown to be of unsound mind (…). Furthermore, the medical assessment must be based on the actual state of mental health of the person concerned and not solely on past events (…).
27.In deciding whether an individual should be detained as a “person of unsound mind,” the national authorities are to be recognized as having a certain discretion since it is in the first place for them to evaluate the evidence adduced before them in a particular case; the Court’s task is to review under the Convention the decisions of those authorities (…).
30.Turning to the facts of the case, the Court notes that the Arnhem Court of Appeal had recourse to a plurality of reports of earlier examinations of the applicant by psychiatrists and psychologists as well as a report by a psychologist and a psychiatrist commissioned while the proceedings were pending before it based on the criminal file and the audio and audio-visual recordings of interrogations. Although the various psychiatrists and psychologists were unable to establish a precise diagnosis, they did express the view that the applicant was severely disturbed, which view the Court of Appeal found reinforced by its own investigation of the case file, of the applicant’s own confused statements especially (…). The Court accepts that, faced as it was with the applicant’s complete refusal to cooperate in any examination of his mental state at any relevant time, the Court of Appeal was entitled to conclude from the information thus obtained that the applicant was suffering from a genuine mental disorder which, whatever its precise nature might be, was of a kind or degree warranting compulsory confinement.’

## Analysis and Discussion

Several issues are relevant here. First of all, it is important to consider what has been said about the use of the DSM classification. In daily practice of behavioral experts, psychiatrists may or may not use the DSM Manual. For instance, an alternative classification is the International Classification of Diseases (ICD classification), which is used in many countries, among which European countries, such as France and Sweden. The fact that a DSM classification would not be decisive for the assessment of a mental disease or mental defect should, therefore, be distinguished from a statement that it does not have to be the behavioral expert who diagnoses the disorder. The profession of psychiatry as an international medical discipline cannot be equated with the DSM classification. Consequently, the—valid—opinion that DSM classification is not decisive does not by itself imply that psychiatric expertise would not be decisive when it comes down to the assessment of a mental illness.

Suppose that without a diagnosis by a psychiatrist and without any other (previous) findings of behavioral experts with regard to the mental capacities of the defendant, the judge would, nevertheless, impose a TBS measure. The TBS setting is a forensic *psychiatric* setting, in which psychiatrists and psychologists work. They determine the plans of treatment and intervention. If the defendant would be admitted to a psychiatric hospital without an underlying diagnosis by a psychiatrist, there might be no valid point of departure for an adequate treatment since no psychiatric disorder was established by the (behavioral) expert in this respect. In other words, there is an inconsistency in the legal reasoning if at some point the disorder is “legal” in nature (and psychiatrists and psychologists were unable to diagnose a disorder), and at another point the person is admitted to a *psychiatric* hospital (5, 6).

Furthermore, without an underlying opinion of a behavioral expert about the mental capacities of the defendant, the judge who would nevertheless establish a psychiatric illness would not be able to specify the disorder. The judge would rule that the criminal responsibility was reduced without explaining how the disorder influences the behavior. Such a legal argument makes it very difficult for a defendant to challenge the court’s decision. How to appeal against such a line of reasoning? What is the framework against which the judge comes to his conclusion, and is this sufficiently clear for the defendant to contest such a conclusion? This is remarkable since, as is clear from the format of questions, such an explanation is required from Dutch behavioral experts: experts should detail the way in which a mental illness impacted on the defendant’s behavior at the time of the crime, and base their judgment of legal insanity (or diminished responsibility) on such an analysis.

One should bear in mind that the law specifically requires that a psychiatrist and a psychologist write a report about the defendant before a TBS measure can be imposed by a court. This strongly suggests, in our view, that the behavioral experts’ *judgment* and *advice* are very important safeguards here. The way in which this requirement is now interpreted is that a psychiatrist and psychologist should *try* to perform an evaluation and even if they were not able to do that, they can *still write a report detailing what they tried*, and that they cannot come to a judgment or advice in terms of criminal responsibility and sanctions which might be imposed by the judge. Clearly in cases in which the behavioral experts disagree, the court may, e.g., choose one of the reports, or ask for a third opinion. This situation, however, differs from cases in which *psychiatrists and psychologists are unable to diagnose a disorder whatsoever*.

Finally, we would like to draw attention to one specific element of the ECtHR judgment, which reads: “Although the various psychiatrists and psychologists were unable to establish a *precise* diagnosis” (23)—while in fact the behavioral experts did not establish any diagnosis. What would be an example of a diagnosis that was “not precise”? Perhaps, an example could be: the defendant was psychotic, but, due to lack of information, the experts are unable to determine the precise nature of the psychosis, e.g., whether it was a psychosis within the context of schizophrenia, bipolar disorder, depression, or substance abuse (all these conditions may lead to/be accompanied by a psychosis). In this case, however, the experts clearly stayed away from making a diagnosis. Furthermore, the ECtHR says that the experts “did express the view that the applicant was severely disturbed.” But does this truly reflect that the experts said that “based on all the information available (…) this reflected the ‘worrying development’ of a young man who had led a detached and antisocial existence, had abused cannabis, and lived in a world of his own (…). The experts O. and R. were unable to supplement it with findings resulting from their own observation”? With its interpretation, the ECtHR somehow appears to overstate what the experts actually reported. In any case, the experts did not explicitly testify: “Even though we are not able to establish a precise diagnosis, in our opinion this defendant is severely disturbed.”

We believe that the criterion of “a true mental disorder must be established before a competent authority on the basis of objective medical expertise” is an important safeguard, and if a “true mental disorder” cannot be established on the basis of “objective medical expertise”—which is psychiatric expertise by nature, not legal expertise—this should have consequences, independent of the reason why a “true mental disorder” could not be established. In fact, this is exactly what the ECtHR formulated: “Where no other possibility exists, for instance, because of a refusal of the person concerned to appear for an examination, at least an assessment by a medical expert on the basis of the file must be sought, failing which it cannot be maintained that the person has reliably been shown to be of unsound mind (…). Furthermore, the medical assessment must be based on the actual state of mental health of the person concerned and not solely on past events.” We feel that these words provide clear guidance. It is now up to the judiciary—including the ECtHR itself—to live up to these words.

In our opinion, the boundaries of the disciplines of the judiciary and behavioral experts, respectively, should not be crossed by one another.^9^ If behavioral experts cannot reach the conclusion—based on their own research and/or based on previous behavioral examinations of the defendant—that the defendant suffers from a mental disease or mental defect, the judge should refrain from an assessment that the defendant nevertheless suffers from a psychiatric illness. The consequence of this line of reasoning is that the judge would indeed be limited with regard to the possibilities of disposal of the criminal case. In case of conviction of the defendant, the judge could not impose a TBS measure. A long(er) imprisonment by way of retribution might be a serious option for a judge.

## Conclusion

Legal insanity is a peculiar element of criminal law, because it brings together two very different disciplines: psychiatry and psychology on the one hand and the law on the other. We conclude that it is crucial—for instance, in terms of legal certainty of (potential) defendants—to clearly distinguish between the responsibilities of the behavioral expert on the one hand and the court on the other. Establishing the presence of a mental illness is an expert’s responsibility which can have far-reaching consequences with regard to the decision of the court concerning the imposition of criminal sanctions. Clearly, if the experts disagree, the court has to make a final judgment, but also within the boundaries of the objective medical expertise presented to the court. We provided several reasons for this position. One who we would like to emphasize is that the defendant should be able to challenge the court’s decision, and therefore the way in which a mental illness is established should be transparent. The ECtHR has provided a valuable legal framework in this respect, relevant to all legal systems falling under its jurisdiction. Its own decision regarding the case we presented, however, seems to depart from that framework. The relevance of the case we presented is, however, not limited to European countries. More generally, it is crucial that legal decisions about a defendant’s illness are founded on the right grounds, in particular where they have far-reaching legal consequences.

## Author Contributions

Both TK and GM: conception of the paper, writing, and revising.

## Conflict of Interest Statement

The authors declare that the research was conducted in the absence of any commercial or financial relationships that could be construed as a potential conflict of interest.

## Footnotes

^1^In jurisdictions in which legal insanity is a defense that has to be raised by the defendant, it may still be possible that the defense is, under certain circumstances, imposed (4). This possibility will not be further considered in this paper.

^2^This is our translation of article 39, based on Ref. (10), p. 74: “Anyone who commits an offence for which he cannot be held responsible by reason of a mental disorder or mental disease is not criminally liable” cf. (11), p. 295: “section 39 of the Dutch Penal Code exempts from punishment the defendant to whom a crime cannot be attributed by reason of a mental disorder.”

^3^According to section 37 paragraph 3 of the Dutch Criminal Code, a psychiatric and psychological evaluation is not required if the defendant refuses to cooperate. In such a case, the behavioral experts report—if possible—about the defendant’s reason not to cooperate and an advice about the desirability and necessity of a security measure must be sought by the judge.

^4^In the Dutch legal system, the behavioural expert gives explicit advice about the level of criminal responsibility. This is not permitted in all legal systems. For instance, in the case of *Washington v. US* (1967) the Court ruled that psychiatrists cannot testify on the ultimate issue.

^5^At the time of the case, the DSM-IV was used, therefore we refer to the DSM-IV.

^6^The DSM-IV-TR (p. xxxii–xxxiii) contains the following cautionary statement with regard to the use of DSM in a forensic context: “When the DSM-IV categories, criteria, and textual descriptions are employed for forensic purposes, there are significant risks that diagnostic information will be misused or misunderstood. These dangers arise because of the imperfect fit between the questions of ultimate concern to the law and the information contained in a clinical diagnosis. In most situations, the clinical diagnosis of a DSM-IV mental disorder is not sufficient to establish the existence for legal purposes of a ‘mental disorder,’ ‘mental disability,’ ‘mental disease,’ or ‘mental defect.’ In determining whether an individual meets a specified legal standard (e.g., for competence, criminal responsibility, or disability), additional information is usually required beyond that contained in the DSM-IV diagnosis.”

^7^The Court of Appeal apparently refers to article 37 § 3 of the Criminal Code, according to the first line of which the second paragraph of article 37 is not applicable if the defendant refuses to cooperate with the behavioral examination.

^8^For several weeks, the defendant has been under observation in a specialized observation clinic, Pieter Baan Center, Utrecht, The Netherlands. From a methodological point of view, if a defendant is not collaborating but he is in custody, an expert might setup an observation setting, and triangulate the ensuing findings with file data. This might yield relevant information for establishing a mental disorder. Yet, what is important in our paper is the Court’s response to the fact that the experts were unable to establish the presence of a mental illness. How the presence can be established by behavioral experts is another matter and may also depend on the legal system.

^9^Confronting scientific and legal criteria in real forensic cases does not only concern the insanity assessment but, at least potentially, also many other kinds of evaluations requested by Courts to psychiatrists and psychologists (e.g., eyewitnesses’ reliability, honest confessions, and suggestibility). Yet, we focus on establishing the presence of a mental illness in a defendant. See also AR Mackor (24).
